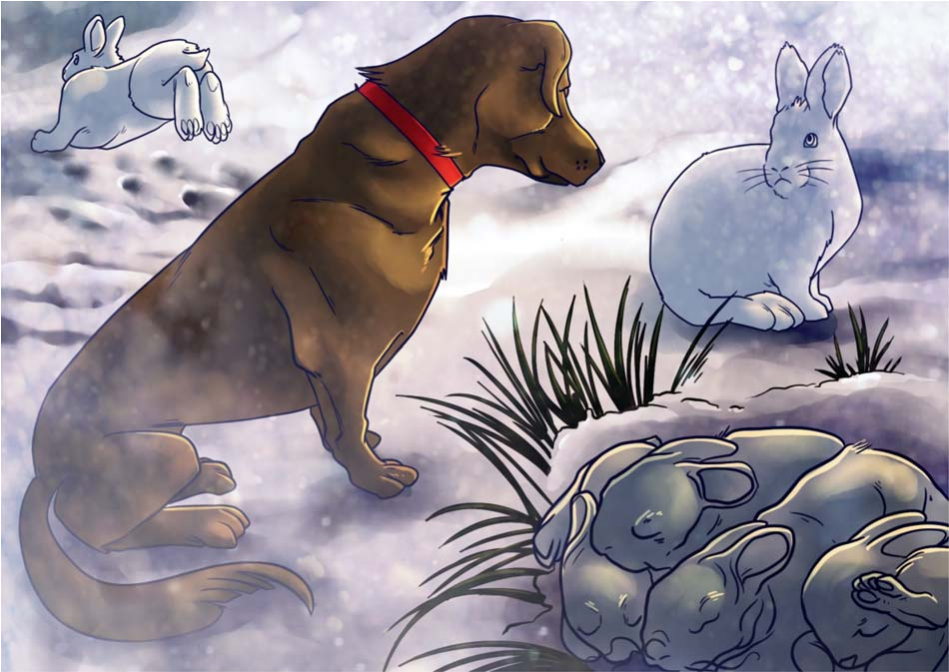# Scared to death? the killer effect of predation risk in snowshoe hares.

**DOI:** 10.1093/conphys/coz029

**Published:** 2019-06-17

**Authors:** Bridie J M Allan

**Affiliations:** Department of Marine Science, University of Otago, Dunedin, New Zealand

Predation is a key theme in ecology. Predators capture, kill and consume their prey, which affects the balance of ecosystems. As ecologists, Lima and Dill succinctly said in 1990, `being killed greatly decreases future fitness’. Seems straightforward, right? However, what happens when predators and prey interact without a lethal event occurring? What exactly does that mean? Imagine hiking through Yellowstone National Park knowing there was a bear on the trail. The prospect of a potential predation event would be quite stressful, right? We would be expending energy activating our hypothalamic–pituitary–adrenal axis and releasing glucocorticoids. What? Simply put, we would be stressed out! However, this energy could be used for growth and fitness instead of the expensive `fight or flight’ stress response.

Described as non-consumptive effects, these occur when predators are nearby. The mere `stress’ of being near a predator can cause prey to change the way they feel, look and behave. This can lead to downstream effects such as changing body shape or getting `stressed out'. For example, some fish produce an eye spot near their tail to divert predatory attacks away from the head. Birds activate stress hormones, leading to changes in parental behaviour impacting offspring survival. Indeed, fear is a powerful response that can lead to maladaptive behaviours. Scared populations of animals are populations that do not thrive. However, little is known about the physiological pressures that non-consumptive effects can exert on mammals.

To test this, MacLeod and colleagues exposed pregnant snowshoe hares (*Lepus americanus*) to dogs that were trained not to bark, whine or harass the hares. The pregnant hares were randomally exposed to the `predator' for a few minutes each time, and the effect of this exposure was documented. If the mothers perceived predation risk during gestation, the survivial of both the offspring and the mothers declined. This suggests that physiological changes associated with mounting a stress response may have led to increased mortality. In fact, simply perceiving predation risk increased cortisol by 214%. In other words, these mothers were stressed out! Not only this, but the effects of releasing stress hormones into the body lasted long after the predator exposure stopped. Offspring from predator-exposed females had lower survival rates from birth to weaning, suggesting predation risk had long-lasting effects on reproduction. Further, elevated stress hormones could prime the offspring, such that they are more susceptible to stress in the future, which may impact the way they behave during risky situations. These non-lethal predator encounters that influence survival of both adults and offspring could lead to problems in snowshoe hare populations.

So, why does this matter? Snowshoe hares play an important role in their ecosystem by maintaining links between the plants that they eat and the carnivores, such as lynx, that prey on them. Changes to snowshow hare populations could disrupt the entire ecosystem. Therefore, understanding how snowshoe hare populations are affected by the presence of predators can tell us how this delicate, ecological balance may change in the future as landscapes change, new predators are potentially introduced or exsisting predators become more protected.

**Cite as:** Kirsty J, Charles J. Krebs, Rudy Boonstra, and Michael J. Sheriff (2018) Fear and lethality in snowshoe hares: the deadly effects of non?consumptive predation risk. *Oikos.* doi:10.1111/oik.04890.

Illustration by Erin Walsh; Email: ewalsh.sci@gmail.com